# BCG Induces PD-1 Upregulation on Circulating CD8^+^ T Cells and IL-6 and IL-8 Secretion *In Vitro*

**DOI:** 10.32604/or.2026.075738

**Published:** 2026-06-16

**Authors:** Gabriela R. Barbosa, Luciana S. B. Dal Col, Caroline C. Bighetto, Maria Carolina X. de Godoy, Marina D. B. P. Campioni, Marcus V. Sadi, Alessandra Gambero, Leonardo O. Reis

**Affiliations:** 1 Immuno-Oncology, Pontifical Catholic University of Campinas (PUC-Campinas), Campinas, SP, Brazil; 2 INCT UroGen, National Institute of Science, Technology and Innovation in Genitourinary Cancer (INCT), Campinas, SP, Brazil; 3 Paulista School of Medicine, Federal University of São Paulo, São Paulo, SP, Brazil; 4 UroScience, State University of Campinas (Unicamp), Campinas, SP, Brazil

**Keywords:** BCG immunotherapy, breast cancer, Toll-like receptors agonists, bladder cancer

## Abstract

**Backgrounds:** Bacillus Calmette-Guérin (BCG) remains the most effective adjuvant therapy for non-muscle-invasive bladder cancer (NMIBC); however, the immunological underpinnings of its efficacy remain incompletely understood. This study aims to elucidate the dual immunomodulatory roles of BCG by integrating systemic and tumor-intrinsic analyses, through determining the systemic effects of BCG instillation on immune checkpoint expression and direct inflammatory response in a previously established *in vitro* tumor model. **Methods:** We investigated systemic and tumor-intrinsic immune responses to BCG. Flow cytometry was used to evaluate immune checkpoint expression on circulating lymphocyte subsets in NMIBC patients (n = 7) at various stages of BCG therapy. In parallel, an *in vitro* model of PD-L1 modulation using breast cancer cell lines (MDA-MB-231 and MCF-7) was stimulated with BCG and TLR agonists, and the secretion of IL-6 and IL-8 was assessed using an ELISA. **Results:** Peripheral immune profiling revealed stable lymphocyte frequencies, but a significant increase in PD-1 expression on CD8^+^ T cells following BCG exposure (*p* = 0.0068), with no significant modulation in CTLA-4 levels. *In vitro*, MDA-MB-231 cells exhibited robust IL-6 secretion upon high-dose BCG stimulation (*p* = 0.0277), whereas MCF-7 cells showed increased IL-8 release (*p* < 0.0001). Other TLR agonists had limited effects. **Conclusions:** BCG induces dual immunomodulation, characterized by PD-1 upregulation in systemic CD8^+^ T cells and the release of pro-inflammatory cytokines from epithelial tumor cells. These findings support the potential of combining BCG with immune checkpoint inhibitors and underscore IL-6 and IL-8 as candidate biomarkers of tumor-intrinsic responsiveness.

## 1 Introduction

Intravesical Bacillus Calmette-Guérin (BCG) immunotherapy remains the most effective adjuvant treatment for non-muscle invasive bladder cancer (NMIBC), harnessing both innate and adaptive immune mechanisms to prevent tumor recurrence and progression [1,2,3,4]. Despite decades of clinical use, the immunological pathways that underpin therapeutic efficacy and those that drive treatment resistance remain incompletely understood. Classically, BCG-induced antitumor immunity has been attributed to the recruitment and activation of immune effector cells, such as neutrophils, macrophages, and T lymphocytes, within the bladder wall [5,6]. However, recent evidence suggests that BCG’s immunomodulatory effects extend beyond direct immune cell activation, potentially involving pro-inflammatory signaling within both tumor and stromal compartments [7,8,9].

Among the key mediators of this inflammatory cascade are the cytokines interleukin-6 (IL-6) and interleukin-8 (IL-8), which regulate leukocyte recruitment, T-cell polarization, angiogenesis, and remodeling of the tumor microenvironment [10,11]. Notably, IL-6 and IL-8 are not exclusively secreted by immune cells: tumor cells themselves can act as immunological amplifiers, responding to pathogen-associated molecular patterns (PAMPs), particularly Toll-like receptor (TLR) agonists, by producing these cytokines. BCG itself functions as a complex TLR agonist, engaging multiple pattern recognition receptors to trigger innate immune signaling [12,13,14]. Since BCG is administered after transurethral resection of bladder tumors, its efficacy relies on modulating immune responses in a tumor-free environment to prevent disease recurrence [4,15].

In this context, our interest lies in elucidating the immunological mechanisms, both systemic and tumor-intrinsic, through which BCG exerts its protective effects. A deeper understanding of these pathways may clarify response variability and guide the development of biomarkers or combination strategies. Unlike prophylactic vaccines, intravesical BCG is a therapeutic immunization given after tumor resection, retraining systemic and mucosal immunity for long-term tumor surveillance rather than transient antibody responses.

The current study aims to elucidate the dual immunomodulatory roles of intravesical BCG by integrating systemic and tumor-intrinsic analyses. First, we assess whether BCG instillation alters the expression of key immune checkpoints, such as Programmed Death-1 (PD-1) and Cytotoxic T-Lymphocyte Associated Protein 4 (CTLA-4), across major circulating lymphocyte subsets, including CD8^+^ T cells, CD4^+^ T cells, NK cells, myeloid-derived suppressor cells (MDSCs), dendritic cells, and naïve T cells. Second, we investigate whether a previously described *in vitro* model for Programmed Death Ligand 1 (PD-L1) modulation [16] responds directly to BCG and other TLR agonists by secreting pro-inflammatory cytokines (IL-6 and IL-8). Through this approach, we aim to uncover novel immune mechanisms and potential biomarkers that may explain interpatient variability and support strategies to optimize BCG efficacy.

## 2 Materials and Methods

### 2.1 Patient Cohort and Sample Collection

After obtaining approval and consent from the Federal University of São Paulo (EPM) ethics committee (number 196/21), peripheral blood samples were collected from patients diagnosed with non-muscle-invasive bladder cancer (NMIBC) undergoing intravesical BCG immunotherapy at the Federal University of São Paulo between September 2021 and April 2023. Inclusion Criteria: NMIBC with the indication for intravesical BCG treatment. Exclusion Criteria: previous BCG treatment or muscle-invasive tumor.

All participants provided written informed consent prior to enrollment, and all procedures involving human subjects were conducted in accordance with institutional and international ethical standards, including the Declaration of Helsinki (1975, revised in 2013). The present analysis is based on a subset of participants from a previously published Phase I randomized clinical trial (NCT04806178) that enrolled 20 patients. All participants received intravesical BCG as per protocol, regardless of prior intradermal priming. For the current immunological analyses, seven consecutive patients with complete or partial longitudinal sampling were included [17].

Specifically, patients were retrospectively stratified into three groups according to cumulative BCG treatment: T0 (unexposed), corresponding to patients prior to therapy initiation; T1 (partially exposed), those who had received two to three weekly instillations; and T2 (fully exposed), defined as individuals who had completed at least five instillations. These timepoints were chosen to reflect progressive immunological engagement with BCG, with T2 representing the minimum exposure threshold for the complete induction course, as supported by clinical practice guidelines [4]. Not all individuals contributed samples at every time point (T0, T1, and T2) due to loss to follow-up or technical limitations during sample collection.

### 2.2 PBMC Isolation and Flow Cytometry

Peripheral blood samples were collected from patients at three defined time points (T0, T1, and T2), corresponding to different stages of BCG exposure. For each sample, 50 μL of fresh whole blood was used for flow cytometric phenotypic analysis of peripheral blood mononuclear cells (PBMCs).

Surface and intracellular markers were stained in a single-tube protocol. The antibody panels (Table 1) were added directly to the fresh blood at the recommended volumes, then incubated for 30 min at room temperature, protected from light. The antibodies used were CD4 (BD Biosciences, 651849, San Jose, CA, USA), CD3 (BioLegend, 300318, San Diego, CA, USA), CD45 (Invritrogen, 69045942, Carlsbad, CA, USA), CD8 (BD Biosciences, 560960), CD45RA (Invitrogen, 61045842), CD62L (BD Biosciences, 341012), CD56 (BioLegend, 343754), CD16 (BioLegend, 980102), CD123 (BioLegend, 306006), HDL-DR (BD Biosciences, 561359), CD11b (BD Biosciences, 557754), CD33 (BD Biosciences, 564588), PD-1 (BD Biosciences, 561273), CTLA-4 (BD Biosciences, 560938).

After staining, 2 mL of PBS (0.1 M) containing 0.5% bovine serum albumin (BSA) and 0.1% sodium azide (NaN_3_) were added, mixed gently, and centrifuged at 540× *g* for 5 min. The supernatant was discarded, and the pellet was gently resuspended.

Intracellular staining was performed using the FIX & PERM^®^ Cell Permeabilization Kit GAS-002 (Nordic MUbio, Netherlands, USA). Cells were fixed with 100 μL of Reagent A (fixative), incubated for 15 min at room temperature, and washed again with 2 mL of PBS + 0.5% BSA + 0.1% NaN_3_. After centrifugation (540× *g*, 5 min), the pellet was resuspended and incubated with 100 μL of Reagent B (permeabilization buffer) for 5 min under the same conditions. After staining, cells were stored at 4°C and protected from light until acquisition. Samples were analyzed on a BD FACSCanto™ II flow cytometer (BD Biosciences, Franklin Lakes, NJ, USA). The gating strategy used for flow cytometry analysis is described in the Supplementary Document (Gating Strategy Used for Flow Cytometry Analysis).

**Table 1:** Markers of major immune cell phenotypes.

Cell Type	Phenotype
T helper	CD4^+^, CD3^+^, CD45^+^
T cytotoxic	CD8^+^, CD3^+^, CD45^+^
T naive	CD3^+^, CD45RA^+^, CD62L^+^
Natural Killer (NK)	CD56^+^, CD3^−^, CD45^+^, CD16^+^
Dendritic Cell (DC)	CD123^+^, HLA-DR^+^, CD45^+^
**Myeloid-Derived Suppressor Cell (MDSC)**	CD11b^+^, CD3^−^, HLA-DR^−^, CD33ˡᵒʷ

Footnote: CD: cluster of differentiation; HLA-DR: Human Leukocyte Antigen-DR isotype.

### 2.3 In Vitro Stimulation and Cytokine Quantification

Previously, cells were treated with Imiquimod (IMQ 37.5 μM, 25 μM, 2.5 μM, 1 μM), Peptidoglycan (PPG 10 μg/mL, 1 μg/mL, 0.1 μg/mL), Lipopolysaccharides from *E. coli* (LPS 1 mg/mL, 100 μg/mL, 10 μg/mL), and BCG (800 μg/mL, 400 μg/mL, 200 μg/mL, 100 μg/mL, 50 μg/mL) to define the highest non-toxic doses [16], selected for cytokine release profiles in response to pattern recognition receptor (PRR) agonists: Imiquimod (IMQ, 25 μM; EMS-Hortolândia, 011895), peptidoglycan (PPG, 10 μg/mL; Sigma, 77140), lipopolysaccharide (LPS, 1 mg/mL; Sigma-Merck, Saint Louis, MO, L2654), or Bacillus Calmette-Guérin (BCG, 200 and 800 μg/mL; Urohipe, Uno Healthcare, Brazil), which were assessed in an *in vitro* model of PD-L1 modulation using MDA-MB-231 and MCF-7 human breast cancer cell lines by quantifying interleukin-6 (IL-6) and interleukin-8 (IL-8) levels in culture supernatants after 48 h of stimulation.

Cell lines were obtained from the Rio de Janeiro Cell Bank, Brazil (BCRJ, codes 0151 and 0182, respectively), and mycoplasma was tracked and STRs validated. MDA-MB-231 cells were maintained in Dulbecco’s Modified Eagle Medium (DMEM; Thermo Fisher Scientific, Waltham, MA, USA) with the addition of 1% antibiotic-antimycotic solution (Gibco, Thermo Fisher Scientific, Waltham, MA, USA). MCF-7 cells were also cultured in DMEM, supplemented with 4500 mg/L glucose, 10% fetal bovine serum (FBS; Gibco, Thermo Fisher Scientific, Waltham, MA, USA), and 1% penicillin-streptomycin (Gibco, Thermo Fisher Scientific, Waltham, MA, USA). Both cell lines were incubated at 37°C in a humidified atmosphere with 5% CO_2_ and routinely subcultured under standard conditions. All culture conditions and the MTT assay, including the full list of concentrations tested and the rationale for selecting these doses, are described in detail in our previous paper [16].

Supernatants from the MTT (3-(4,5-dimethylthiazole-2-yl)-2,5-diphenyl tetrazolium bromide) assays were collected, clarified by centrifugation, and stored at −80°C until analysis. BD OptEIA™ Human IL-6 ELISA Set 550799, and Human IL-8 ELISA Set 555244 (BD Biosciences, San Diego, CA), according to the manufacturer’s instructions. All conditions were tested in duplicate, and cytokine concentrations were interpolated from standard curves within the assay’s linear range.

### 2.4 Statistical Analysis

Given the relatively small, cross-sectional, and non-paired nature of the dataset, all immunophenotyping parameters, including immune cell frequencies and checkpoint molecule expression, were analyzed by treating T0, T1, and T2 as independent groups. From a feasibility and interpretability standpoint, cross-sectional analyses prioritize validity and interpretability over potentially misleading longitudinal inferences, allowing for consistent analytical treatment across serial samples and enabling comparisons of snapshots over time without claiming causality or temporal effects that the data are not powered to support.

Data were summarized using median and interquartile range (IQR). Comparisons were made using Kruskal-Wallis tests with Dunn’s post hoc corrections. Significance was defined as *p* < 0.05. Analyses and graphs were generated in GraphPad Prism v10, GraphPad Software, LLC, Boston, MA, USA.

## 3 Results

### 3.1 Flow Cytometric Analysis of Circulating Immune Subsets during BCG Treatment

All seven included NMIBC patients were diagnosed with pT1 high-grade pure urothelial bladder cancer, having undergone full transurethral resection of bladder tumor (TURBT) with no residual tumor during the current study, and no history of previous therapy with intravesical BCG. A minimum of 5 patients were analyzed at each time point (T0, T1, and T2).

The immunophenotypic analysis of peripheral blood immune cells across the three stages of BCG exposure revealed mostly stable cell frequencies, with selective changes in immune checkpoint expression.

The frequency of CD4^+^ T cells remained consistent over time, with medians of 39.5% [IQR: 30.8–45.6] at baseline (T0, n = 5), 42.1% [IQR: 39.5–46.6] at mid-treatment (T1, n = 4), and 40.7% [IQR: 30.9–41.6] at treatment completion (T2, n = 4) (*p* = 0.3200). Similarly, CD8^+^ T cell frequencies did not significantly vary across timepoints: (T0, n = 4) 13.2% [IQR: 10.1–19.4], (T1, n = 5) 19.2% [IQR: 11.3–20.8], and (T2, n = 4) 19.6% [IQR: 8.7–36.4] (*p* = 0.3875). NK cells showed a non-significant fluctuation: 15.1% [IQR: 11.0–20.2] at T0 (n = 4), 12.4% [IQR: 10.8–14.9] at T1 (n = 6), and 23.9% [IQR: 16.2–33.9] at T2 (n = 4) (*p* = 0.3214). MDSC frequencies exhibited a downward trend over time—(T0, n = 4) 1.61% [IQR: 0.99–2.83], (T1, n = 4) 0.49% [IQR: 0.27–1.78], and (T2, n = 4) 0.28% [IQR: 0.14–1.41]—though without reaching statistical significance (*p* = 0.1512).

A transient increase in dendritic cells was observed at T1 (n = 5) (1.31% [IQR: 0.44–1.74]), compared to 0.88% [IQR: 0.53–1.89] at T0 (n = 4) and 0.58% [IQR: 0.16–0.81] at T2 (n = 4) (*p* = 0.1297). Naïve T cells showed a modest, non-significant increase across treatment: (T0, n = 4) 2.02% [IQR: 1.20–4.55], (T1, n = 5) 2.48% [IQR: 0.68–2.75], and (T2, n = 2) 4.82% [IQR: 2.23–5.41] (*p* = 0.1168) (Supplementary S1: Changes in the frequency of circulating immune cell populations during intravesical BCG immunotherapy).

With respect to immune checkpoint dynamics, PD-1 expression increased significantly on CD8^+^ T cells over the course of BCG therapy (*p* = 0.0068), whereas no significant modulation was detected in CD4^+^ T cells (*p* = 0.2089), NK cells (*p* = 0.7944), MDSCs (*p* = 0.9131), dendritic cells (*p* = 0.8618), or naïve T cells (*p* = 0.9164). CTLA-4 expression remained stable across all evaluated subsets: CD4^+^ (*p* = 0.9959), CD8^+^ (*p* = 0.7058), NK (*p* = 0.1036), MDSCs (*p* = 0.8151), DCs (*p* = 0.4770), and naïve T cells (*p* = 0.3070) (Supplementary S2: PD-1 and CTLA-4 Expression in CD8^+^ T Cells During BCG Therapy).

**Figure 1:**
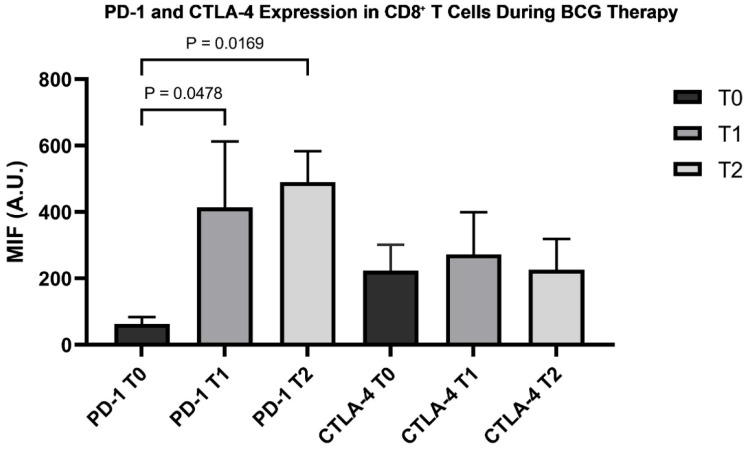
Programmed Death-1 (PD-1) and Cytotoxic T-Lymphocyte Associated Protein 4 (CTLA-4) Expression in CD8^+^ T Cells During Bacillus Calmette-Guérin (BCG) Therapy. MIF: mean fluorescence intensity for PD-1 and CTLA-4 in different times (T0, T1 and T2) in CD8^+^ cells (A. U.: arbitrary units; T: time).

**Figure 2:**
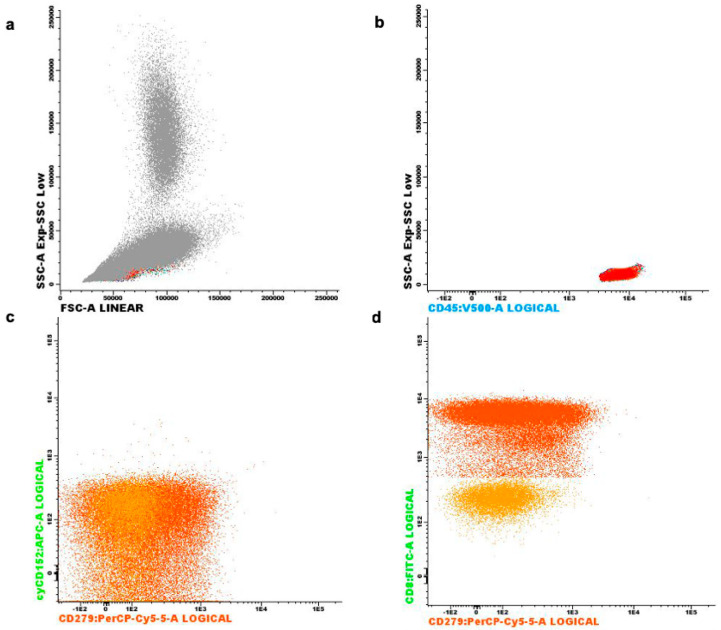
(**a**): Representative dot plot of Forward Scatter (FSC-A) versus Side Scatter (SSC-A). (**b**): Representative dot plot of CD45 expression in T-Lymphocytes. (**c**): Representative dot plot of CD152 versus CD279 expression in CD8^+^ T cells, represented in orange. (**d**): Representative dot plot of CD297 expression in CD8^+^ T cells, in orange.

Collectively, these findings suggest that BCG immunotherapy exerts selective effects on peripheral immune modulation, most notably by upregulating PD-1 on CD8^+^ T cells following full induction (Fig. 1 and Fig. 2).

Mean fluorescence intensity (MFI) of PD-1 and CTLA-4 on peripheral CD8^+^ T cells at baseline (T0), mid-treatment (T1), and after completion of BCG therapy (T2). PD-1 expression significantly increased at T1 and T2 compared to T0 (*p* = 0.0478 and *p* = 0.0169, respectively), while CTLA-4 levels remained stable throughout the treatment course. Data are presented as median frequencies and interquartile ranges across treatment time points. Statistical analysis was performed using the Kruskal–Wallis test followed by Dunn’s multiple comparisons test.

### 3.2 Cytokine Production in Breast Cancer Cell Lines upon Innate Immune Stimulation

**Figure 3:**
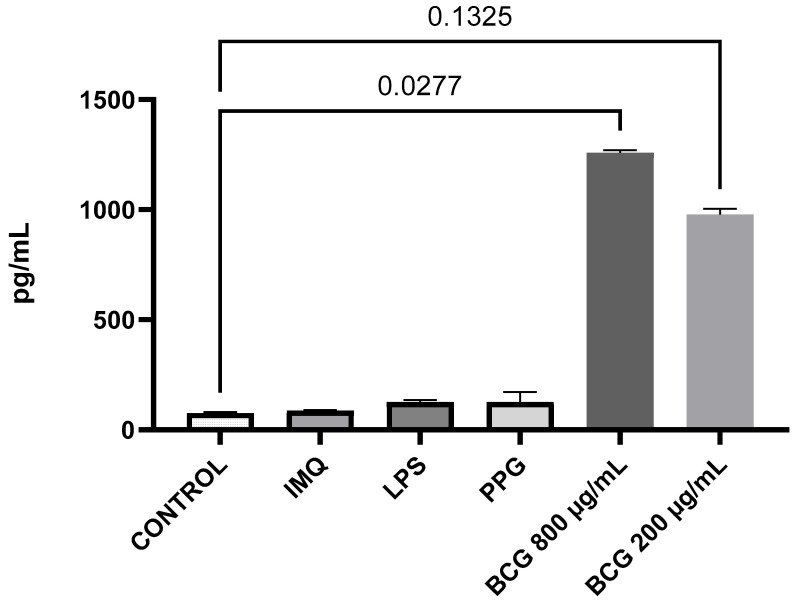
IL-6 Secretion by MDA-MB-231 Cells in Response to Bacillus Calmette-Guérin (BCG) and Toll-like receptors agonists imiquimod (IMQ), peptidoglycan (PPG), and lipopolysaccharide (LPS).

MDA-MB-231 cells: IL-6 secretion varied significantly across conditions (*p* = 0.0011, Kruskal–Wallis’s test). Median IL-6 levels (pg/mL) ranged from 75.4 [IQR 71.96–78.79] in untreated controls to 1257.7 [IQR 1248.2–1267.2] following high-dose BCG 800 μg/mL, which was the only condition to induce a statistically significant increase versus control (*p* = 0.0277). Low-dose BCG 200 μg/mL elicited a non-significant elevation, with a median of 977.6 [IQR 958.9–996.2] (*p* = 0.1325). Other stimuli, including imiquimod (86.4 [IQR 83.7–89.2]), LPS (125.6 [IQR 118.3–132.9]), and peptidoglycan (125.3 [IQR 92.4–158.2]), did not significantly alter IL-6 secretion (*p* = 0.8 for all comparisons) (Fig. 3).

IL-8 production in MDA-MB-231 cells showed no significant differences between groups (*p* = 0.0841), despite elevated levels following LPS (2107.5 [IQR 1477.9–2737.1]), imiquimod (1603.4 [IQR 1524.7–1682.1]), and peptidoglycan (1467.1 [IQR 1284.6–1649.6]) stimulation. High- and low-dose BCG induced moderate IL-8 release, with medians of 1174.2 [IQR 919.6–1428.7] and 905.8 [IQR 761.4–1050.2], respectively, but did not reach statistical significance.

**Figure 4:**
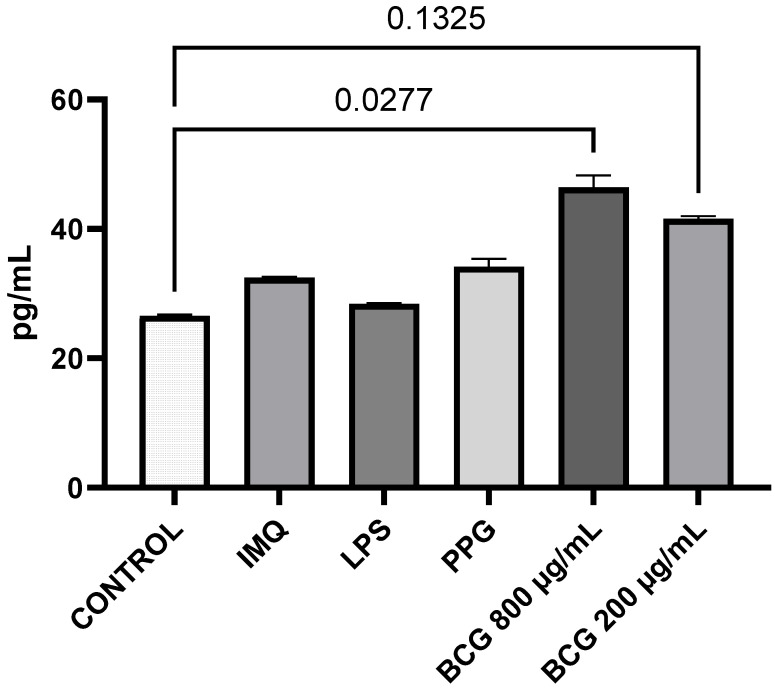
IL-8 Secretion by MCF-7 Cells in Response to Bacillus Calmette-Guérin (BCG) and Toll-like receptor agonists imiquimod (IMQ), peptidoglycan (PPG), and lipopolysaccharide (LPS).

MCF-7 cells: IL-6 levels remained low across all conditions (*p* = 0.1962). Untreated controls showed a median of 5.7 [IQR 2.8–8.5] pg/mL. Imiquimod induced a modest increase (55.3 [IQR 43.2–67.3]), followed by peptidoglycan (20.4 [IQR 8.1–32.6]), LPS (11.4 [IQR 9.6–13.3]), and high- and low-dose BCG (12.9 [IQR 12.9–12.9] and 14.6 [IQR 11.8–17.4], respectively), but none reached significance. Conversely, IL-8 production in MCF-7 cells differed significantly between conditions (*p* < 0.0001). High-dose BCG induced a statistically significant increase compared to control (46.4 [IQR 45.1–47.7] vs. 26.6 [IQR 26.4–26.7]; *p* = 0.0277), whereas low-dose BCG elicited a non-significant elevation (41.6 [IQR 41.2–41.9]; *p* = 0.1325). Imiquimod (32.5 [IQR 32.4–32.6]), LPS (28.4 [IQR 28.4–28.5]), and peptidoglycan (34.2 [IQR 33.3–35.0]) did not significantly affect IL-8 secretion (Fig. 4).

Together, these results demonstrate a differential cytokine response profile between breast cancer subtypes, with BCG exerting a more robust IL-6–driven pro-inflammatory effect in triple-negative MDA-MB-231 cells, while selectively enhancing IL-8 secretion in luminal MCF-7 cells. A dose-response pattern was observed between 200 and 800 μg/mL BCG.

## 4 Discussion

Although BCG remains the cornerstone of NMIBC immunotherapy, its downstream immunological effects are incompletely defined [12,18]. This study aimed to delineate both systemic and tumor-intrinsic responses to BCG, focusing on PD-1 expression in circulating immune subsets and inflammatory signaling in an *in vitro* model of PD-L1 modulation using MDA-MB-231 and MCF-7 human breast cancer cell lines [16]. The resulting data reveal a dual immunomodulatory effect, engaging cytotoxic T cells in the periphery while simultaneously triggering pro-inflammatory responses in epithelial tumor cells.

The immunophenotypic analysis of peripheral immune subsets revealed largely stable frequencies across BCG treatment, including CD4^+^ and CD8^+^ T cells, NK cells, MDSCs, dendritic cells, and naïve T cells. None of these populations exhibited statistically significant shifts, indicating that BCG does not significantly alter the number of circulating immune cells.

Instead, its effects appear functionally selective, particularly through a qualitative modulation of cytotoxic T cells. CD8^+^ T cells emerged as the most responsive subset, while changes in CD4^+^ T cells, NK cells, dendritic cells, and MDSCs were minor or inconsistent. This pattern suggests that BCG’s systemic immunomodulatory effects are preferentially routed through cytotoxic effector pathways, as also observed in preclinical models [6,19]. The lack of CTLA-4 modulation across all subsets likely reflects its predominant role during early T-cell priming in lymphoid tissues rather than active regulation in the peripheral compartment [20].

Regarding immune checkpoint dynamics, we observed a significant increase in PD-1 expression on circulating CD8^+^ T cells after full-course BCG, whereas PD-1 levels remained unchanged in CD4^+^ T cells, NK cells, MDSCs, dendritic cells, and naïve T cells. Similarly, CTLA-4 expression was stable across all evaluated subsets. These data highlight PD-1 as the main regulatory checkpoint engaged in peripheral cytotoxic T cells in response to BCG, consistent with its role as a critical immune brake that prevents overactivation and potential immunopathology [21]. Chakra et al. revised the role of PD-1 as a compensatory checkpoint induced by chronic antigenic stimulation during BCG therapy, modulating T-cell function within a cytokine-rich inflammatory milieu [22].

Although elevated PD-1 expression is classically associated with T cell exhaustion in chronic antigen exposure and the tumor microenvironment, its expression is highly context- and time-dependent and intricately regulated by different genetic and epigenetic programs. During acute immune activation, PD-1 is transiently upregulated as a physiological regulatory mechanism and does not necessarily indicate functional exhaustion. In the context of BCG exposure, increased PD-1 expression on CD8^+^ T cells likely reflects recent activation and engagement in an effective antitumor response rather than terminal dysfunction [23].

On the other hand, tumor immune evasion is a dynamic process characterized by the development of an immunosuppressive microenvironment, with the PD-1/PD-L1 pathway playing a multifaceted regulatory role [24]. Our data reinforce the central role of PD-1 in systemic immune regulation post-BCG and support the rationale for combining BCG with PD-1/PD-L1 blockade to overcome adaptive immune resistance and improve clinical outcomes. Confirming the clinical relevance of our mechanistic results, 2 of 3 clinical trials published in 2025 demonstrated a significant improvement in disease-free survival when combining BCG with immune checkpoint inhibitors in patients with non-muscle-invasive bladder cancer [25,26,27].

There is also reliable evidence that BCG can treat other cancers beyond urothelial cancer, mainly breast cancer [28,29]. Adding a breast cancer *in vitro* protocol represents an innovative approach to better understand BCG mechanisms and support the next steps toward broader clinical application. Our results are consistent with the previously described synergistic effect of Bacillus Calmette-Guérin and anti-PD-L1 in an orthotopic triple-negative breast cancer mouse model [29].

The current study demonstrates that MDA-MB-231 cells secreted substantial IL-6 in response to high-dose BCG exposure, whereas MCF-7 cells predominantly secreted IL-8. These differences likely reflect intrinsic variations in PRR responsiveness, NF-κB/STAT3 signaling thresholds, and cytokine gene accessibility [30,31]. This pattern supports previous evidence that mesenchymal-like (i.e., MDA-MB-231) tumor cells exhibit a more inflammatory secretory profile than epithelial-like (i.e., MCF-7) tumor cells [32,33]. This duality may shape local immune dynamics and influence responses to BCG immunotherapy.

Normal breast epithelial cells primarily produce IL-6 and IL-8 as major cytokines. Breast cancer tissues exhibit elevated IL-8 levels compared with normal tissue, which correlates with angiogenesis [34,35]. The IL-8 secreted by tumor cells promotes endothelial cell proliferation, survival, and matrix metalloproteinase (MMP) production [36]. Additionally, breast cancer cell lines produce IL-6, with estrogen receptor (ER)-positive cells secreting lower levels than ER-negative cells; IL-6 is known to induce proliferation and foster a more aggressive phenotype in ER-positive cells [37].

Clinical studies have reported elevated urinary and tumor IL-6/IL-8 levels following BCG therapy, supporting the notion that the cytokine patterns identified here may have patient-level correlates and potential as predictive biomarkers. Urothelial cells, which line the urinary tract, display robust expression of membrane-bound and cytosolic pattern recognition receptors (PRRs), including TLR2, TLR4, TLR5, TLR9, as well as NOD1 and NOD2, enabling rapid detection of bacterial components and activation of NF-κB- and MAPK-dependent signaling pathways that drive the production of pro-inflammatory mediators such as IL-6 and IL-8 [10,11,38,39]. Breast epithelial cells exhibit a more context-dependent PRR profile; they express selected TLRs (including TLR3, TLR4, and TLR9) and produce IL-6 and IL-8 [31,32], as demonstrated in the current study. Both the bladder and the mammary gland maintain low microbial biomass environments with robust innate immune defenses despite ductal communication with the external environment, limiting sustained microbial exposure under physiological conditions [40,41].

Taken together, our data support a model in which systemic immune activation and tumor-intrinsic signaling converge to establish a complex immunological ecosystem following BCG exposure. PD-1 upregulation on CD8^+^ T cells may reflect not only direct activation by microbial antigens, but also indirect regulation mediated by tumor-derived IL-6 and IL-8. Recent evidence from prostate cancer patients shows that IL-6 is not only elevated in tumors but also correlates with disease progression and immune profile shifts, suggesting its potential as a biomarker to guide therapy decisions [42]. In future studies, IL-6 (alongside IL-8) should be tested to identify which patients are more likely to respond or fail to BCG immunotherapy, thereby paving the way for more personalized interventions [38,39,43].

This work uniquely integrates *in vivo* systemic checkpoint modulation with *in vitro* tumor-intrinsic cytokine profiling. Critically, the strength of our study lies in capturing the dynamic functional PD-1 induction in CD8^+^ T cells, indicating that checkpoint engagement reflects treatment-driven immune activation induced by BCG, rather than static checkpoint expression [44].

Limitations should be acknowledged. This study is a subanalysis of a Phase I randomized trial in which all 20 participants received intravesical BCG. For the current immunological investigation, only seven patients with available blood samples were included. Not all contributed samples at every time point (T0, T1, T2), which accounts for variability in group sizes. Importantly, the objective here was not to compare trial arms but to evaluate longitudinal immune responses to intravesical BCG independently of prior randomization. The relatively small sample size and cross-sectional, non-paired design may limit the detection of subtle immunological changes and constrain generalizability.

Future studies that expand sample size and use paired-sample analysis to enhance statistical power are necessary to validate the current results. Also, the clinical relevance of IL-6 and IL-8 (e.g., predicting BCG response and monitoring therapeutic efficacy), as well as the functional significance of changes in PD-1 and PD-L1 expression in the clinical setting, are beyond the scope of the current study. It is worth noting that the functional significance of PD-L1 is poorly understood and does not correlate with tumor response. The KEYNOTE-045 trial demonstrated improved overall survival with pembrolizumab (anti-PD-1 antibody) compared with chemotherapy in patients with advanced urothelial carcinoma regardless of PD-L1 status, further supporting the notion that PD-L1 expression is not a reliable standalone predictive biomarker [45].

Although not bladder-derived, the *in vitro* model of PD-L1 modulation using MDA-MB-231 and MCF-7 human breast cancer cell lines [16] reveals conserved epithelial inflammatory circuits including PRR expression and cytokine release, potentially shared with urothelial carcinoma, as we have shown after intravesical BCG treatment in an autochthonous bladder cancer animal model [24]. These results align with current findings and are poised to inform future studies on the issue.

In conclusion, current findings suggest that BCG immunotherapy elicits coordinated immune remodeling across systemic and epithelial compartments, characterized by the upregulation of PD-1 in CD8^+^ T cells and the secretion of pro-inflammatory cytokines by tumor cells. This interconnected signaling may sustain inflammation while maintaining immune regulation.

While current results are in line with observations from preclinical models and are biologically plausible [46], the limited sample size and the exploratory nature of the analysis raise concerns regarding the robustness of the statistical significance. Future studies should confirm and expand on the hypothesis-generating nature of the findings, including the greater sensitivity of specific peripheral blood immune cells to certain checkpoint engagement under BCG exposure, and BCG’s unique ability to induce IL-6 and IL-8 in tumor cells compared to diverse PRR agonists. Concurrently, urine tumor DNA to detect minimal residual disease and further disentangle the contributions of surgery and immunotherapy to disease control should be added to the definition of tumor-free after transurethral resection of bladder tumor (TURBT) in future studies [47,48].

Deciphering these interplays is essential for identifying predictive biomarkers and for developing rational combination strategies to enhance the therapeutic efficacy of BCG in oncology.

## Data Availability

The authors confirm that the data supporting this study’s findings are available within the article.

## Supplementary Materials

The supplementary material is available online at https://www.techscience.com/doi/10.32604/or.2026.075738/s1.
