# Supplementary material for: BCG Induces PD-1 Upregulation on Circulating CD8+ T Cells and IL-6 and IL-8 Secretion In Vitro
Source: Oncol Res. 2026 Jun 16;34(7):20. doi: 10.32604/or.2026.075738 (PMC13292045; doi:10.32604/or.2026.075738)
Supplement: Supplementary file 1 [file OncolRes-34-75738-s001.zip › TSP_OR_75738-Supplementary_Material.pdf]

Supplementary Document:

Gating Strategy Used for Flow Cytometry Analysis

To ensure reproducibility and transparent interpretation, flow cytometry data were analyzed using a standardized, sequential gating strategy. All analyses were performed on compensated data, using fluorescence-minus-one (FMO) controls where applicable.

Step 1. Exclusion of debris

Initial gating was performed on **Forward Scatter (FSC-A) versus Side Scatter (SSC-A)** to exclude debris and non-cellular events, and the main cell population was selected based on size and granularity.

```
SSC-A
│
│     ●●●●●  ← Cells
│   ●●●●●●●
│ ●●●●●●●●●
│
│ x x x x x     ← Debris
└──────────────────────────────────── FSC-A
```

Step 2. Doublet discrimination

To ensure analysis of single cells only, **doublets were excluded** using FSC-A versus FSC-H (or FSC-W).

```
FSC-H
│     ● Single cells
│    ●
│   ●
│
│  x x        ← Doublets
└──────────────────────────────────── FSC-A
```

Step 3. Live/dead cell discrimination

Dead cells were excluded using a **viability dye** (Live/Dead Fixable dyes). Only **viability dye–negative cells** were included.

```
Viability Dye
│  x x x x      ← Dead cells (excluded)
│
│ ●●●●●●●●●      ← Live cells (kept)
└────────────────────────────────────
```

Step 4. Lineage or pan-population identification

Live singlets were gated on a **lineage or pan-cell marker**, depending on the experiment (e.g., CD45$^+$ leukocytes).

Marker (e.g., CD45)
| ●●●●● ← CD45$^+$ cells
|
| x x x ← CD45$^-$ cells
└────────────────────

Step 5. Subpopulation identification

Within the parent population, sequential gates were applied to define specific subsets using combinations of markers. Example for immune profiling:

- CD3$^+$ T cells
- CD4$^+$ and CD8$^+$ T cells
- Further functional or activation markers (e.g., PD-1)

CD8
| ●●● ← CD8$^+$
|
| ●●●●● ← CD4$^+$
└──────────────────── CD4

Step 6. Functional or phenotypic analysis

Functional markers (e.g., checkpoint molecules - PD-1) were quantified within the final gated populations. Gates were defined using **FMO controls** to minimize subjective thresholding.

Functional Marker
| ●● ← Positive
|
| ●●●●●● ← Negative
└────────────────────

Quality Control and Reproducibility Measures

- Instrument performance was monitored using standardized calibration beads.
- Compensation matrices were generated using single-stained controls.
- FMO controls were used for all multicolor panels to define positive populations.
- Gating templates were applied uniformly across all samples.
- Analysis was performed using FlowJo, v11.0.2

S1. Changes in the frequency of circulating immune cell populations during intravesical BCG immunotherapy.

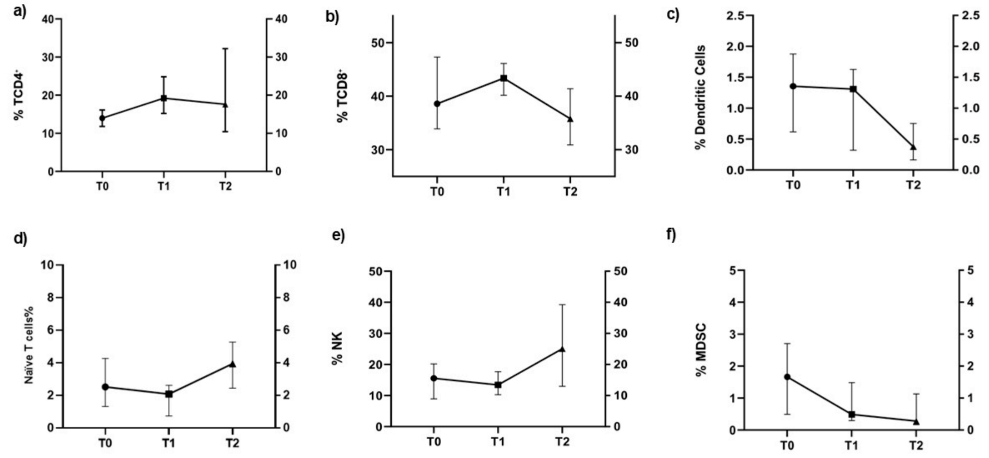

Panels A–F show the distribution of CD4^{+} T helper cells (A), CD8^{+} cytotoxic T cells (B), dendritic cells (C), naïve T cells (D), natural killer (NK) cells (E), and myeloid-derived suppressor cells (MDSCs) (F) in peripheral blood at three treatment timepoints: T0 (unexposed), T1 (partially exposed), and T2 (fully exposed) of BCG induction course. Data are presented as medians with corresponding interquartile ranges (25th to 75th percentiles).

S2. PD-1 and CTLA-4 Expression in CD8⁺ T Cells During BCG Therapy

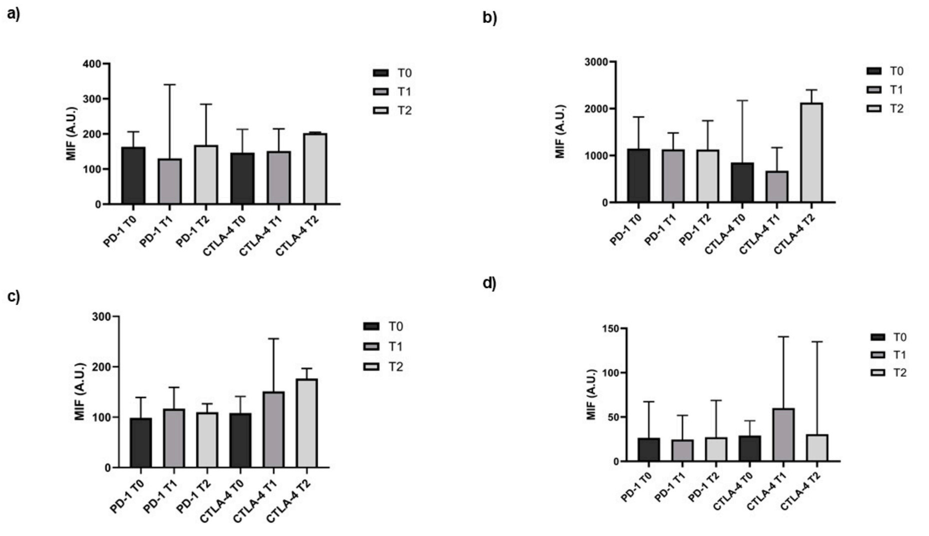

Mean fluorescence intensity (MFI) of PD-1 and CTLA-4 on dendritic cells (A), naïve T cells (B), natural killer (NK) cells (C), and myeloid-derived suppressor cells (MDSCs) D) at baseline (T0), mid-treatment (T1), and after completion of BCG therapy (T2). Data are shown as median frequencies and interquartile ranges across treatment timepoints.
